# Safe RESIDential Environments? A longitudinal analysis of the influence of crime-related safety on walking

**DOI:** 10.1186/s12966-016-0343-4

**Published:** 2016-02-16

**Authors:** Sarah Foster, Paula Hooper, Matthew Knuiman, Hayley Christian, Fiona Bull, Billie Giles-Corti

**Affiliations:** Centre for the Built Environment and Health, School of Sport Science, Exercise & Health and School of Earth & Environment, The University of Western Australia (M087), 35 Stirling Highway, Crawley, WA 6009 Australia; School of Population Health, The University of Western Australia, 35 Stirling Highway, Crawley, WA 6009 Australia; McCaughey VicHealth Centre for Community Wellbeing, Melbourne School of Population Health, University of Melbourne, Melbourne, Australia

**Keywords:** Safety from crime, Perceptions, Longitudinal, Walking, Built Environment, Adults

## Abstract

**Background:**

Numerous cross-sectional studies have investigated the premise that the perception of crime will cause residents to constrain their walking; however the findings to date are inconclusive. In contrast, few longitudinal or prospective studies have examined the impact of crime-related safety on residents walking behaviours. This study used longitudinal data to test whether there is a causal relationship between crime-related safety and walking in the local neighbourhood.

**Methods:**

Participants in the RESIDential Environments Project (RESIDE) in Perth, Australia, completed a questionnaire before moving to their new neighbourhood (*n =* 1813) and again approximately one (*n =* 1467), three (*n =* 1230) and seven years (*n =* 531) after relocating. Self-report measures included neighbourhood perceptions (modified NEWS items) and walking inside the neighbourhood (min/week). Objective built environmental measures were generated for each participant’s 1600 m neighbourhood at each time-point, and the count of crimes reported to police were generated at the suburb-level for the first three time-points only. The impact of crime-related safety on walking was examined in SAS using the Proc Mixed procedure (marginal repeated measures model with unrestricted variance pattern). Initial models controlled for demographics, time and self-selection, and subsequent models progressively adjusted for other built and social environment factors based on a social ecological model.

**Results:**

For every increase of one level on a five-point Likert scale in perceived safety from crime, total walking within the local neighbourhood increased by 18.0 min/week (*p =* 0.000). This relationship attenuated to an increase of 10.5 min/week after accounting for other built and social environment factors, but remained significant (*p =* 0.008). Further analyses examined transport and recreational walking separately. In the fully adjusted models, each increase in safety from crime was associated with a 7.0 min/week increase in recreational walking (*p =* 0.009), however findings for transport walking were non-significant. All associations between suburb-level crime and walking were non-significant.

**Conclusions:**

This study provides longitudinal evidence of a potential causal relationship between residents’ perceptions of safety from crime and recreational walking. Safety perceptions appeared to influence recreational walking, rather than transport-related walking. Given the popularity of recreational walking and the need to increase levels of physical activity, community social and physical environmental interventions that foster residents’ feelings of safety are likely to increase recreational walking and produce public health gains.

**Electronic supplementary material:**

The online version of this article (doi:10.1186/s12966-016-0343-4) contains supplementary material, which is available to authorized users.

## Background

Numerous studies have tested the prevailing assumption that neighbourhood crime will deter residents from engaging in physical activity, yet findings to date are equivocal and inconsistent [1–3]. In some studies, higher crime rates or perceptions of crime have been associated with lower levels of physical activity [4–7], whereas others report no association [8, 9], or even counterintuitive positive associations [10, 11]. In part, this could be explained by the considerable variation in the measures used to capture ‘safety from crime’. While these diverse measures embody similar concepts, they do not necessarily represent the same construct [10]. For instance, studies have documented limited agreement between *subjective* and *objective* measures of crime [12–15], suggesting they may capture different elements of the neighbourhood environment [9, 15]. Furthermore, there is considerable scope within *subjective* measures of crime-related safety, including the distinction between judgements (or cognitive) assessments of crime and emotional (or affective) responses to crime. To illustrate – residents may perceive higher levels of crime in their local area, but if the crime they perceive does not make them feel unsafe or fearful (i.e., an emotional response), it may not affect their behaviour [1]. Given the lack of consensus in the literature to date on whether actual crime or the perception of crime impacts on physical activity, there is merit in focusing on and comparing studies that adopt similar measures of crime-related safety.

Another criticism of the literature on safety from crime and physical activity is the dearth of evidence from longitudinal studies. Few prospective or longitudinal studies have explored the impact of crime or perceptions of crime on physical activity [16–18] [19, 20], and no clear pattern has emerged. For example, Kerr et al. [18] found no relationship between changes in adults’ perceptions of crime-safety and walking for leisure or transport in Chicago, USA, but did find an increase in the local murder rate correlated with decreases in transport walking. In contrast, our longitudinal study in Perth, Australia, found strong evidence supporting a causal relationship between fear of crime and walking, where each increase in fear (on a five-point Likert scale) was associated with a 22 min/week decrease in total walking in the local neighbourhood [17]. However, it is worth highlighting again that the different crime-related safety measures applied in these studies may contribute to the conflicting findings (i.e., exposure measures included objective crime rates, cognitive assessments of crime, and emotional responses to crime).

While our previous study [17] found that *fear of* crime was sizable deterrent to walking, relatively few studies to date have applied similar ‘emotional’ measures (i.e., fear or anxiety about crime) [1, 21–23]. Most studies use measures that are best conceptualised as judgements (or cognitive assessments) of *safety from* crime, such as the Neighbourhood Environment Walkability Scale (NEWS) [24]. One benefit of using the NEWS crime-safety measure is that its’ wide scale use in international studies facilitates comparison [7, 25–27]. For example, a recent meta-analysis using cross-sectional data from 12 countries, providing considerable environmental and cultural variability, found higher perceptions of safety from crime (measured with NEWS) were associated with increased odds of recreational walking [7].

Other reasons could also account for inconsistencies in the evidence base, including a geographic mismatch between the exposure measure and the outcome [1]. Crime-related safety measures routinely focus on the local neighbourhood, but the outcome measures of walking or physical activity are rarely measured at the same scale or setting. Further, it is plausible that only physical activities conducted in neighbourhood public spaces would be constrained by perceptions of local crime and safety. Participation in other physical activities that contribute to a respondent’s total physical activity level (e.g., gym workouts, dance classes, gardening and other home based activities) are less likely to be influenced by neighbourhood crime levels or perceptions of safety [1]. To date, few studies have measured both exposure and outcome variables for the same geographic area [1].

This study tests whether there is a causal relationship between residents’ perceptions of safety from crime (measured with NEWS items) and walking within the local neighbourhood in Perth, Western Australia. Using a longitudinal study design, we focused specifically on the safety-walking relationship, with progressive adjustment for other neighbourhood attributes that could mediate the relationship between safety and walking (i.e., built environment, social cohesion, aesthetics).

## Methods

### Sample and data collection

The RESIDential Environments (RESIDE) Project is a longitudinal natural experiment of people building houses and relocating to 73 new housing developments across Perth, Western Australia. All people building new homes in the study areas were invited to participate by the state water authority following the land transfer transaction (response rate 33.4 %). They completed a self-report questionnaire before they moved into their home (*n =* 1813), and on three occasions after relocation at approximately one (*n =* 1467), three (*n =* 1230) and seven years (*n =* 531). At each time-point, objective physical environmental measures were generated in Geographic Information Systems (GIS) for each participant’s individual ‘neighbourhood’. RESIDE was approved by The University of Western Australia’s Human Research Ethics Committee (#RA/4/1/479) and is described elsewhere [28]. All participants provided informed consent to participate in the study.

Overall, the study sample was older and slightly more affluent than the wider Perth metropolitan area population [29], reflecting a population group able to purchase a new home. Given the sample and suburban setting, our study neighbourhoods were relatively safe, with little evidence of serious antisocial behaviour [30] and negligible serious crime [12].

### Measures

Outcome variables: Walking was measured using the Neighbourhood Physical Activity Questionnaire (NPAQ), which has acceptable test-retest reliability (ICC ≥ 0.82), and distinguishes the location (i.e., inside versus outside the neighbourhood) and purpose of walking (i.e., transport versus recreational) [31]. Walking outcomes included minutes/week of walking in the local neighbourhood for: (1) transport; (2) recreation; and (3) total walking.

Independent variables: Perceived safety from crime was measured using a modified version of the Neighbourhood Environment Walkability Scale (NEWS) [24]. Items included: (1) there is a lot of petty crime in my local area (reversed); (2) there is a lot of major crime in my local area (reversed); (3) the level of crime in my local area makes it unsafe to go on walks during the day (reversed); (4) the level of crime in my local area makes it unsafe to go on walks at night (reversed); and (5) I would feel safe walking home from a bus or train stop at night. Factor analyses indicated that these items all loaded highly on one factor (Cronbach’s alpha = 0.77). Participants rated each item on a Likert scale (1 = strongly disagree to 5 = strongly agree), and these were averaged to create a composite scale for each time-point (higher scores indicate greater safety from crime). Objective crime was supplied by The Western Australia Police for the calendar years corresponding with completion of the baseline, year 1 and year 3 questionnaires only. Crimes were limited to those committed against the person in public space (e.g., threats, disorderly behaviour, assault, robbery).

### Adjustment variables

Demographic and self-selection variables included gender, age, marital status, education, household income, and the importance of safety from crime as a reason for neighbourhood selection at baseline (measured using the scale: 1 = not at all important to 5 = very important).

Perceived environment variables included other modified NEWS items [24] that captured resident’s perceptions of neighbourhood presentation and upkeep. Items were modified by substituting ‘local area’ for ‘neighbourhood’ and other wording changes to better reflect the local vernacular (e.g., footpaths instead of sidewalks, 50 kph instead of 30 mph). NEWS items have established reliability and validity [32–34], and the modified items were re-assessed for test-retest reliability and deemed reliable (Fleiss, 1981). Items (rated 1 = strongly disagree to 5 = strongly agree) were combined into scales capturing ‘neighbourhood aesthetics’ (Cronbach’s alpha = 0.76) and ‘traffic hazards’ (Cronbach’s alpha = 0.63), with a single item measuring ‘street lighting’.

Social cohesion was measured using a modified version of the Neighbourhood Cohesion Index (NCI), which measures psychological sense of community [35]. Our scale comprised 16 five-point Likert items (1 = strongly disagree to 5 = strongly agree), including items such as: I feel like I belong to this neighbourhood; I visit with my neighbours in their homes; and I feel that there is a bond between me and other people in my neighbourhood. The complete list of items is documented elsewhere [36]. All items had moderate to high test-retest reliability and high internal consistency (Cronbach’s alpha = 0.93). Higher values indicate greater sense of community within the neighbourhood context [35].

Objective built environment measures included: (1) street connectivity (i.e., count of ≥ three-way intersections); (2) residential density (i.e., ratio of the land area in residential use to the number of residential dwellings); and (3) local destinations (i.e., the count of local shopping and service destinations derived from a commercial database). These measures were calculated from data acquired from the Western Australian state government (i.e., The Western Australian Land Information Authority and Western Australian Department of Planning for road centreline and property cadastre data) and a commercial database (i.e., SENSIS Yellow Pages for local destinations). A new data extraction was acquired at each time-point (i.e., four data extractions over the study period); with the year of the data chosen to temporally match to the year of survey completion as closely as possible. The built environment measures were recalculated in GIS at each time-point for the 1600 m road network service area around each participant’s home. This represents the *maximum* distance a person could walk in 15 min (using the speed 6 km/h), and is consistent with a return trip being 30 min of exercise, as per physical activity guidelines [37–39].

### Statistical analysis

Models were fitted in SAS software (version 9.4) using Proc Mixed. We applied a marginal repeated measures model with an unrestricted variance pattern across time points that used all available data from the four time-points for each person. The primary models estimated the overall effect of safety from crime (i.e., the combined between-person (or cross-sectional) and within person (or longitudinal) effect). Additional models were run that decomposed the safety from crime measure into between-person and within-person measures to separately estimate the cross-sectional and longitudinal effect. A series of multivariable models examined the associations between perceived safety from crime and the walking outcomes with progressive adjustment for: (1) demographic and self-selection factors (i.e., age, gender, marital status, education, household income, and the importance of safety from crime to neighbourhood selection at baseline), (2) built environment factors (i.e., residential density, street connectivity, local destinations); (3) social environmental factors (i.e., perceived social cohesion); and (4) neighbourhood perceptions (i.e., aesthetics, traffic, street lighting). These models were rerun with crimes reported to police (i.e., an ‘objective’ suburb-level crime measure) substituted for perceived safety from crime (these analyses were limited to all available data from three RESIDE time-points – baseline, year 1 and year 3).

Finally, additional models tested for effect modification (i.e., by gender, age group, educational status) and whether there was a non-linear relationship between perceived safety from crime and walking.

## Results

At baseline the mean age of participants was 40 years, 60 % were female, 82 % were married or living with a partner, and safety from crime rated very highly in their choice of new neighbourhood (Table 1). Table 2 shows the study variables at each time-point. Most notable changes occurred between baseline (i.e., before participants moved house) and year 1 (i.e., after relocating to new residential developments). For example, most participants moved from an area with good access to local destinations to an area with relatively few destinations. The number of destinations increased in year 3 and year 7, but did not return to baseline levels. In contrast, most neighbourhood perceptions improved between baseline and year 1, and generally remained constant at subsequent time-points. For instance, safety from crime increased between baseline and year 1, and remained stable for year 3 and year 7. However, aesthetics was an exception, whereby perceptions improved between baseline and year 1, and then declined to return to baseline levels for the following time-points.

**Table 1:** Baseline socio-demographic characteristics of the cohort (*n =* 1831)

Characteristic	%
Age^a^	40.0 (11.9)
Gender	
Male	40.3
Female	59.7
Income	
Less than $50,000	25.9
$50,000 to $69,999	25.0
$70,000 to $89,999	23.2
$90,000 or more	25.9
Education	
Secondary or less	39.4
Trade / Apprenticeship / Certificate	37.6
Bachelor degree or higher	22.9
Marital Status	
Partner	81.5
No Partner	18.5
Importance of safety to neighbourhood selection^a^	4.4 (0. 8)

^a^Value is expressed as mean (standard deviation). Importance of safety from crime to neighbourhood selection was measured using a Likert scale (1 = not at all important; 5 = very important)

**Table 2:** Study variables at each time point

	Baseline (*n =* 1813)	Year 1 (*n =* 1467)	Year 3 (*n =* 1230)	Year 7 (*n =* 531)
Variable	Mean (SD)	Mean (SD)	Mean (SD)	Mean (SD)
Built environment				
Residential density^a^	15.1 (8.0)	12.7 (5.4)	14.1 (5.2)	14.3 (4.1)
Street connectivity^b^	61.5 (18.0)	73.8 (25.7)	78.8 (26.0)	82.3 (27.4)
Local destinations^c^	52.6 (72.7)	15.6 (36.7)	20.7 (49.0)	25.8 (38.3)
Social environment				
Social cohesion	3.0 (0.6)	3.6 (0.6)	3.5 (0.6)	3.5 (0.6)
Perceptions				
Aesthetics	3.4 (0.7)	3.6 (0.6)	3.4 (0.6)	3.4 (0.6)
Traffic hazards	2.6 (0.8)	2.2 (0.6)	2.3 (0.6)	2.4 (0.6)
Street lighting	3.1 (1.0)	3.5 (0.9)	3.4 (0.9)	3.5 (0.9)
Safety from crime				
Perceived safety from crime	3.4 (0.8)	3.8 (0.6)	3.7 (0.6)	3.7 (0.6)
Crimes reported to police^d^	88.4 (86.9)	71.6 (92.1)	76.6 (85.9)	-
Walking (min/week)				
Total walking	96.3 (139.3)	109.4 (178.5)	121.1 (214.0)	109.9 (139.5)
Walking for recreation	68.7 (98.4)	89.0 (112.8)	90.0 (127.5)	87.6 (121.4)
Walking for transport	26.6 (57.8)	19.8 (50.2)	25.6 (68.5)	27.8 (69.6)

^a^Ratio of the land area in residential use to the number of residential dwellings

^b^Count of three (or more) way intersections

^c^Count of local destinations (all retail and service destinations)

^d^Crimes committed against the person in public space (e.g., threats, disorderly behaviour, assault, robbery) summarised by suburb (data unavailable at Year 7)

Suburb-level crimes reported to police also reduced after participants relocated to their new areas, but rose slightly with more time in their new neighbourhood. The mean values for crime reported to police show that, on average, participants lived in safe areas (e.g., at baseline there was an average of 88.4 crimes in these suburbs for the calendar year), although there was considerable variation among participants. Notably, there was only a very weak correlation between suburb-level crime and participants perceptions of safety from crime (all values <0.2).

The associations between safety from crime and walking (i.e., recreational, transport and total walking) were examined with progressive adjustment for other attributes (Table 3). Safety from crime was associated with total walking (Model 1), where for each increase in safety (i.e., one level on a five-point Likert scale), walking inside the local neighbourhood increased by 18 min/week (*p =* 0.000). This association was consistent despite additional adjustment for built environment factors (Model 2), and although it attenuated slightly with further adjustment for social cohesion (Model 3) and neighbourhood perceptions (Model 4), it remained significant. In the final model, each increase of one level in safety from crime was associated with a 10 min/week increase in total walking inside the local neighbourhood (*p =* 0.008).

**Table 3:** Relationship between perceived safety from crime and walking inside the neighbourhood (min/week)

Variable	Model 1 Demographics		Model 2 Built environment		Model 3 Social cohesion		Model 4 Perceptions	
	Estimate (SE)	p	Estimate (SE)	p	Estimate (SE)	p	Estimate (SE)	p
Total walking	18.04 (3.50)	**0.0001**	18.77 (3.50)	**0.0001**	13.53 (3.61)	**0.0002**	10.54 (3.97)	**0.0079**
Walking for recreation	13.51 (2.36)	**0.0001**	13.70 (2.37)	**0.0001**	10.25 (2.46)	**0.0001**	7.01 (2.70)	**0.0096**
Walking for transport	3.18 (1.27)	**0.0127**	3.56 (1.27)	**0.0051**	1.38 (1.31)	0.2922	0.68 (1.44)	0.6378

Proc Mixed marginal model with unrestricted variance pattern

Model 1 adjusts for age, gender, income, education, marital status, importance of safety from crime to neighbourhood selection and time

Model 2: Model 1 + residential density, street connectivity and local destinations

Model 3: Model 2 + perceptions of neighbourhood social cohesion

Model 4: Model 3 + perceptions of aesthetics, traffic and street lighting

Bold denotes significant p-value

Additional models examined walking for recreation and walking for transport separately. The association between safety and recreational walking was consistent with the pattern for total walking. In Model 1, each increase of one level in perceived safety from crime was associated with a 13 min/week increase in recreational walking (*p <* 0.001). This remained constant in Model 2, but attenuated slightly in Models 3 and 4. In the final model (Model 4) each increase of one level in safety from crime was associated with a 7 min/week increase in recreational walking (*p =* 0.01). In contrast, the effect size was smaller and statistical significance weaker for transport walking. In Model 1, each increase of one level in safety from crime was associated with a 3 min/week increase in transport walking (*p =* 0.013), however this association fully attenuated with further adjustment in the subsequent models.

These same models were rerun with a decomposed predictor in order to separate the ‘between’ (or cross-sectional effect) from the ‘within person’ (or longitudinal) effect. The contrast tests comparing the between and within person estimates were non-significant in all cases (all *p >* 0.30), justifying the presentation of the overall results only.

In contrast to the results for perceived safety from crime, the relationship between crimes reported to police (i.e., an ‘objective' crime measure) and walking was non-significant across all models for all outcomes (see Additional file 1). Additional models revealed no evidence of curvature in the relationship between perceived safety from crime and walking, nor any effect modification by the main socio-demographic variables (e.g., age, gender, marital status, education).

## Discussion

For this sample of suburban residents, we found longitudinal evidence that perceived safety from crime was associated with increased time spent walking in the local neighbourhood, and more specifically with recreational walking. However, the strong findings in the initial models attenuated with the inclusion of social cohesion and other neighbourhood perceptions (i.e., aesthetics, lighting, traffic), suggesting interventions that build stocks of social cohesion and improve neighbourhood presentation and upkeep could minimise the impact of crime perceptions on walking. This aligns with Crime Prevention through Environmental Design Principles (CPTED) and the traditional neighbourhood planning codes (e.g., new urbanism) that recognise that street-level design factors and neighbourhood planning can influence the quality of the public realm [30, 40], provide opportunities for social interactions and connections [41], and enhance feelings of safety [12, 42, 43].

Few longitudinal or prospective studies have examined the impact of perceived safety from crime on walking or physical activity. Two Dutch studies examined the impact of area-level safety on physical activity, and found that fear of crime [19] and feelings of unsafety [20] at baseline were associated with levels of physical activity at follow-up, however neither found evidence supporting a causal relationship (i.e., changes in fear/safety between time-points were not associated with physical activity). Another prospective study found the perception of higher crime at baseline was associated with fewer minutes of moderate to vigorous physical activity among women at follow-up [16]. However, it appears that only two studies have used a longitudinal design to explicitly test whether changes in individual-level perceptions of crime-related safety influence changes in walking. Of these, one had null findings [18], whereas our earlier analysis from the RESIDE study found that increases in fear of crime were a significant deterrent to walking [17]. Consistent with this, the current study results were in the anticipated direction, adding weight to the hypothesis that perceptions of crime-related safety are a significant population-level barrier to walking.

To date, there has been little consensus in the literature on whether safety concerns inhibit physical activity [1–3], however, the findings from RESIDE reveal a consistent association between subjective measures of crime and walking, regardless of whether the independent crime safety variable is conceptualised as cognitive or affective [4, 17]. We previously suggested that the distinction between cognitive and affective measures might account for the mixed findings, and that affective measures (i.e., fear of crime) could have a bigger impact on behaviour [1, 17]. Our results from the longitudinal analyses suggest that this may still be a valid hypothesis – for example, in the current study, perceived *safety from crime* (i.e., a cognitive measure) was associated with a 10 min/week change in total walking, however our previous results found that changes in *fear of crime* (i.e., an affective measure) were associated with a 22 min/week change in total walking [17]. Thus, while cognitive measures of crime-safety influenced participants walking, the effect size was larger for the affective fear of crime measure.

Nevertheless, it is worth reflecting upon why safety from crime is so pertinent to walking for our sample of suburban residents. First, this study focused on walking within the local neighbourhood, a behaviour that typically occurs in public space and is therefore more likely to be impacted by feelings of safety than other physical activities conducted in private space. Further, both the crime-safety and walking measures were specific to the same geographic location (i.e., the 10–15 min walk from home), meaning there was a good spatial match between the predictor and outcome (i.e., perceptions of safety in the local neighbourhood are less likely to impact on physical activity undertaken elsewhere) [1, 44]. Thus, measurement issues did not obscure any potential relationship between crime-related safety and walking.

Second, in this study the RESIDE sample were sufficiently affluent to purchase a house and land package in relatively safe new suburban developments [45]. This highlights an apparent contradiction, as although the results showed that perceptions of crime-safety influenced walking, our participants lived in neighbourhoods with relatively minimal crime [12, 45]. This suggests there may be something intrinsic to our somewhat middle class sample that makes them more sensitive to crime. Indeed, there is some support in the literature for the notion that the middle classes have heightened concerns about crime and safety. Taylor et al. [46] suggests residents’ reactions to physical disorder (e.g., litter, graffiti and vandalism) differ by area-level income. In higher income neighbourhoods disorder can be uncommon and is disregarded, in lower income neighbourhoods residents may have more pressing priorities or hold external agencies (e.g., government, landlords) responsible for neglecting the area, but in middle income neighbourhoods, where many homeowners experience mortgage stress, residents may have a heightened awareness of neighbourhood deterioration and/or crime. The higher homeownership rates make it less plausible that ‘outsiders’ are to blame and residents may question their investment [46]. Further, Farrall et al. [47] characterise those who have minimal experience of crime, live in relatively safe, well maintained and socially cohesive areas, as ‘anxious’. This group may not experience the ‘sharp end’ of crime, but nevertheless they appear to be preoccupied with it, perhaps as an outlet for other, more generalised worries about life (p.243) [47]. These descriptions seem fitting for our sample of homeowners. Indeed, the importance of neighbourhood safety appears pervasive for our participants because at baseline they rated their reasons for selecting their new neighbourhood, and safety from crime ranked second only to affordability [28].

In this study, we separately examined the longitudinal relationship between suburb-level crime and walking, and found no associations. Our results indicated that residents’ perceptions of safety from crime are a more powerful deterrent to walking than actual crime. However, the correlations between these ‘objective’ measures of crime and participants perceptions of crime were very weak, supporting the contention that ‘objective’ and ‘subjective’ measures of crime may represent different constructs [15]. Even so, it is worth noting that we examined crime at a relatively coarse geographic scale, and there is cross-sectional evidence that crime measured at more proximate spatial scales may have stronger associations with physical activity [48]. Further, we focused on crimes against the person in public space, which may be under-reported and does not capture the relatively minor offences that can act as a visual reminder of local problems (e.g., graffiti, vandalism, litter, drug paraphernalia). Previous RESIDE work identified a potentially causal relationship between residents perceptions of disorder and their perceptions of crime [45]. Finally, RESIDE participants lived in relatively safe neighbourhoods, and it is possible that the levels of crime were simply too low to impact on behaviour.

The strengths of this study include the use of a ‘cognitive’ measure of safety from crime (rather than an ‘emotional’ or ‘affective’ measure) to ensure the findings relate to the broader international literature that uses similar safety measures. Our analyses also has a very large sample, using all available data on every RESIDE participant (i.e., 1813 at baseline, 1467 at year 1, 1230 at year 3, and 531 at year 7). Further, in this longitudinal analysis, we separately estimated the cross-sectional and longitudinal associations (via the decomposed measure) and found them to be similar. This underscores the robustness of our finding, as the association holds both cross-sectionally and longitudinally. Notably, the estimated longitudinal effect is not (unlike the cross-sectional estimate) subject to bias from any (measured or unmeasured) time-constant selection factors and other confounders [49]. This longitudinal effect (as compared to the cross-sectional effect) is also much less likely to be due to reverse causality.

Nonetheless, this study has several limitations. First, our findings may be specific to middle class suburban residents and not generalizable to other settings and populations. However, the results are certainly relevant to the residents in the suburban greenfield developments that continue to unfold in many developed countries such as Australia and the USA. Second, both our main exposure measure and outcome were self-report, potentially introducing a same-source bias [50]. Self-report walking measures are also predisposed to over-reporting and recall biases, however this allowed us to focus on walking conducted within the neighbourhood, which was paramount [51]. Further, if the self-reporting bias is in the same direction on all occasions, it would be less of a concern in our longitudinal analyses. Third, we defined the (subjective) local neighbourhood as a 10–15 min walk from home, and the objective built environment was operationalised as the 1600 m road network distance from home. However, there is increasing awareness that arbitrarily defined neighbourhoods may bear little resemblance to what a person actually perceives their neighbourhood to be [52, 53]. Finally, walking for recreation was more prevalent among our participants than transport walking, and this may have contributed to the small, attenuating association identified for perceived safety from crime and transport walking.

## Conclusions

This study contributes new evidence supporting a causal association between perceptions of safety from crime and walking. We found that for every one level increase in perceived safety from crime, participants total walking inside the neighbourhood increased by 10 min, even after accounting for other social and built environmental factors. These findings indicate that interventions designed to improve perceptions of safety could increase walking levels – yet this suggestion comes with an important caveat as our study was conducted in new suburban developments that typically experience low levels of crime and are generally well kept and presented. Our findings also support our hypothesis that the distinction between cognitive and affective crime-safety measures that might account for the mixed findings in the literature. While we found that perceived safety from crime was important, affective measures (i.e., fear or anxiety about crime) may ultimately have a bigger impact on behaviour. Future studies in more diverse settings and populations are necessary to corroborate our findings, using spatially matched predictor and outcome measures, to support the notion that improving neighbourhood social cohesion and upkeep is a viable means to enhance safety perceptions and increase walking levels.

## Additional file:

Additional file 1:
**Relationship between crimes reported to police** **and walking inside the neighbourhood (min/week).** (DOC 29 kb)
