# Supplementary material for: Safe RESIDential Environments? A longitudinal analysis of the influence of crime-related safety on walking
Source: Int J Behav Nutr Phys Act. 2016 Feb 16;13:22. doi: 10.1186/s12966-016-0343-4 (PMC4755004; doi:10.1186/s12966-016-0343-4)
Supplement: Additional file 1: — Relationship between crimes reported to police and walking inside the neighbourhood (min/week). (DOC 29 kb) [file 12966_2016_343_MOESM1_ESM.doc]

**Supplementary Table 1: Relationship between crimes reported to police^1^** and walking inside the neighbourhood (min/week)

| **Variable** | **Model 1**  **Demographics** | | **Model 2**  **Built environment** | | **Model 3**  **Social cohesion** | | **Model 4**  **Perceptions** | |
| --- | --- | --- | --- | --- | --- | --- | --- | --- |
|  | **Estimate (SE)** | **p** | **Estimate (SE)** | **p** | **Estimate (SE)** | **p** | **Estimate (SE)** | **p** |
|  |  |  |  |  |  |  |  |  |
| Total walking | -0.23 (0.33) | 0.4887 | -0.62 (0.32) | 0.0551 | -0.44 (0.31) | 0.1587 | -0.30 (0.30) | 0.3208 |
| Walking for recreation | -0.17 (0.20) | 0.4104 | -0.24 (0.21) | 0.2592 | -0.11 (0.20) | 0.5942 | 0.00 (0.20) | 0.9796 |
| Walking for transport | 0.12 (0.12) | 0.2912 | -0.04 (0.12) | 0.7231 | 0.02 (0.11) | 0.8442 | 0.04 (0.11) | 0.6831 |

Proc Mixed marginal model with unrestricted variance pattern

Model 1 adjusts for age, gender, income, education, marital status, importance of safety from crime to neighbourhood selection, time and clustering within suburb

Model 2: Model 1 + residential density, street connectivity and local destinations

Model 3: Model 2 + perceptions of neighbourhood social cohesion

Model 4: Model 3 + perceptions of aesthetics, traffic and street lighting

^1^Associations are for every additional 10 crimes against the person in public space reported to police
